# Supplementary material for: MetaRibo-Seq measures translation in microbiomes
Source: Nat Commun. 2020 Jun 29;11:3268. doi: 10.1038/s41467-020-17081-z (PMC7324362; doi:10.1038/s41467-020-17081-z)
Supplement: Supplementary file 10 — Supplementary Data 7 [file 41467_2020_17081_MOESM10_ESM.zip › File2/Confidence_High_Taxonomy/293995_out.krona.html]

Javascript must be enabled to view this page.

members
magnitude
magnitudeUnassigned
count
unassigned
taxon
rank

293995\_out

14

2
superkingdom
14

976
phylum
14

14
class
200643

171549
14
order

12
family
171552

577309
12
genus

species

SRS015782\_contig\_number\_30086SRS017307\_contig\_number\_4399SRS043841\_contig\_number\_contig-100\_34452.34453SRS045004\_contig\_number\_9534SRS047044\_contig\_number\_5359SRS063370\_contig\_number\_9404SRS064232\_contig\_number\_432SRS077294\_contig\_number\_12989SRS077589\_contig\_number\_635SRS101376\_contig\_number\_223SRS148159\_contig\_number\_32109SRS893252\_contig\_number\_6525
12
454154

2
family
1853231

2
genus
283168

28118
2

SRS063985\_contig\_number\_26938SRS893187\_contig\_number\_305
species
